# Case Report: Effective management of a Meige syndrome patient with subthalamic stimulation-induced dyskinesia through timed stimulation programming of different contacts

**DOI:** 10.3389/fnhum.2026.1743270

**Published:** 2026-01-22

**Authors:** Dawei Meng, Haihang Sun, Ning Wang, Zonghui Fu, Lin Wang

**Affiliations:** 1 Department of Neurosurgery, Aviation General Hospital, Beijing, China; 2 Department of Neurosurgery, Meihekou Central Hospital, Meihekou, China

**Keywords:** deep brain stimulation, Meige syndrome, stimulation-induced dyskinesia, subthalamic nucleus, timed stimulation

## Abstract

**Introduction:**

Meige syndrome is a rare adult-onset segmental dystonia characterized by blepharospasms and oromandibular dystonia. Deep brain stimulation (DBS) of the subthalamic nucleus (STN) is an established treatment, but it can lead to stimulation-induced dyskinesia (SID) in some patients. Refractory SID in Meige syndrome after STN-DBS is clinically challenging. We report a case of a Meige syndrome patient who developed refractory SID following STN-DBS and was successfully managed using a novel timed-stimulation programming strategy employing different contacts.

**Case description:**

A 47-year-old female with a two-year history of Meige syndrome developed refractory SID after the treatment of STN-DBS. Various programming strategies were attempted, including monopolar stimulation, interleaved stimulation, bipolar stimulation et al., but none achieved a balance between symptom control and SID. A novel approach involving timed alternation between ventral contacts (contacts 3 and 7) and dorsal contacts (contacts 4 and 8) was implemented. The stimulation was gradually programmed, the duration of ventral stimulation was increased while decreasing dorsal stimulation. Eventually, the patient achieved significant symptom improvement without SID. The reconstruction of the volume of tissue activated (VTA) revealed that this stimulation strategy likely modulates the neural circuits of pallidothalamic fibers (PTF) to suppress SID.

**Conclusion:**

This case demonstrates that this noval timed stimulation programming can effectively manage refractory SID in Meige syndrome patients, offering a viable alternative when conventional methods fail. The findings suggest that PTF stimulation plays a key role in SID suppression, and this strategy warrants further investigation in larger cohorts.

## Introduction

Meige syndrome is a rare adult-onset segmental dystonia characterized by blepharospasms and oromandibular dystonia and may be accompanied by symptoms affecting the neck and upper extremities. Deep-brain stimulation (DBS), especially that of the subthalamic nucleus (STN) DBS, is a well-established treatment for Meige syndrome. Patients have been reported to experience 58.9–74% improvement in BFMDRS motor scores after STN-DBS (Zhan et al., 2018; Xie et al., 2024). However, many patients may experience stimulation-induced dyskinesia (SID) after STN-DBS. STN-DBS-induced dyskinesia occurred in 32.6% of Parkinson’s disease (PD) patients, with 7% exhibiting persistent symptoms beyond 6 months (da Candeias Silva et al., 2025). This phenomenon has also been observed in 19–31% of Meige syndrome patients after STN-DBS (Liu et al., 2021; Wang et al., 2021). Various strategies have been used to reduce SID, but some patients still experience persistent refractory SID. We report a case in which a Meige syndrome patient with refractory SID after STN-DBS was successfully managed with novel strategies, such as timed stimulation programming for different contacts. This method, guided by a detailed analysis of the patient’s individual programming process and clinical response, had not been previously described for the treatment of SID.

## Case description

A 47-year-old female was admitted to the Neurosurgical Department of our hospital with a two-year history of Meige syndrome, which was characterized by blepharospasms and oromandibular and neck dystonia (Supplementary Video 1). The total Burke–Fahn–Marsden Dystonia Rating Scale (BFMDRS) score was 29. The motor subscore was 26 (eyes: 8; mouth: 8; speech/swallowing: 4; neck: 6), and the disability subscore was 3 (speech: 2; feeding: 1). The patient had no response to oral medications, such as benzhexol or clonazepam, and only a partial response to botulinum toxin injections. She subsequently underwent bilateral STN-DBS (electrode: L301; implantable pulse generator: G102RZ; PINS Medical Co., Ltd., Beijing, China).

Initial programming was performed 1 month after surgery. During initial programming, the patient quickly developed slight SID in the right arm, approximately half an hour after monopolar stimulation of contacts 1, 2 and 3 of the left STN lead. She was programmed with monopolar stimulation of contact 3 (left STN) and contact 7 (right STN), which was accompanied by a slightly tolerated right arm SID. One month after stimulation, her blepharospasms significantly improved, and the oromandibular dystonia underwent partial remission. However, the symptoms of SID were deteriorated into rapid jerks in the right arm, right leg, left leg and trunk. Her stimulation parameters were subsequently switched to monopolar stimulation with more dorsal contact 4 (left STN) and contact 8 (right STN), resulting in complete resolution of her symptoms for 1 week. Afterward, her symptoms reappeared and gradually worsened. To address this, the voltage was gradually increased to 3.8 V to improve her condition. This adjustment led to an improvement in her blepharospasms, but there was only a slight improvement in her oromandibular and cervical dystonia. Importantly, no additional SID was observed with these parameters. Afterward, we changed the parameters to monopolar stimulation with contacts 3 and 7 at a voltage of 1.3 V and gradually and slowly increased the voltage to 1.5 V during a period of 2 months, but she still experienced severe SID (Supplementary Video 2), and her symptom improvement was not as good as before. We subsequently applied interleaved stimulation; however, she experienced unbearable dizziness and gait disability, and her symptom improvement remained unsatisfactory. We subsequently used bipolar and double monopolar stimulation. These strategies were also incapable of achieving an equilibrium between the management of symptoms and SID. We advised the patient to switch to monopolar stimulation with contacts 3 and 7 and more slowly increase the voltage starting from the point without SID. The patient disagreed because she experienced very minimal symptom improvement when the stimulation parameter was set in the absence of SID.

Twenty-five months after surgery, we analyzed the patient’s programming process and observed that her symptoms improved after monopolar stimulation with contacts 3 and 7, but SID appeared approximately half an hour after stimulation initiation and gradually worsened within several days. Hence, we experimented with a new stimulation protocol, which included timed alternation between monopolar stimulation with contacts 3 and 7 (parameter 1) and contacts 4 and 8 (parameter 2). We set the stimulation duration of parameter 1 within 2 h and then switched back to parameter 2 for 3 h. The stimulation duration allocation of parameter 1 and parameter 2 was determined based on the patient’s individual clinical response during prior programming sessions. At the beginning of timed stimulation, to ensure that SID could be monitored without becoming severe, we limited the duration of parameter 1 to no more than 2 h per interval, summing to a total of 10 h per day. Then parameter 2 was found to provide superior symptom relief compared to parameter 1 at the beginning of timed stimulation. Therefore, parameter 2 was strategically allocated to cover critical periods of the patient’s daily activities-specifically around breakfast (6:00–8:00), lunch (10:00–13:00), dinner (15:00–18:00), and before sleep (20:00–23:00)-with each session lasting approximately 3 h. This schedule aimed to ensure optimal symptom control during these important functional periods. This approach allowed the patient to achieve improvement without experiencing severe SID. Excitingly, no significant SID was observed in this patient upon the application of parameter 1. Simultaneously, we slowly increased the voltage of parameter 1 from 1.5 V on the basis of the occurrence of SID. During the timed alternation protocol, when the voltage for parameter 1 was increased to 1.85 V (28 months post-operation), the patient exhibited better symptom improvement with parameter 1 than with parameter 2. We subsequently gradually decreased the duration of parameter 2 during meal and pre-sleep intervals while progressively increasing the duration of parameter 1. Thirty-two months after surgery, the parameters were changed to full time continuous stimulation with parameter 1 and a voltage of 2.0 V. With these parameters, the patient’s symptoms significantly resolved. Excitingly, during this process, the patient only experienced mild SID in the right arm, which gradually disappeared without the occurrence of any other SID manifestations. The specific parameter adjustment process is detailed in Table 1. It has been 41 months since the surgery. The patient continues to receive full-time stimulation with parameter 1 and a voltage of 2.0 V. Until the last programming (32 months postoperatively), the patient experienced significant alleviation of symptoms without SID for 9 months, as noted at the latest follow-up (41 months postoperatively) (Supplementary Video 3). The total BFMDRS score was 2. The motor subscore was 2 (eyes: 1; mouth: 1), and the disability subscore was 0.

**Table 1:** The stimulation parameter during the programming course.

Post-operation time (m)	Parameter							
	Left side				Right side			
	Contact	Amplitude (V)	Pulse width (us)	Frequency (Hz)	Contact	Amplitude (V)	Pulse width (us)	Frequency (Hz)
1 m	C+ 3−	1.5	60	130	C+ 7−	1.5	60	130
2 m	C+ 4−	2.5	60	130	C+ 8−	2.5	60	130
6 m	C+ 4−	3.8	90	120	C+ 8−	3.8	90	120
7 m	C+ 3−	1.3	60	120	C+ 7−	1.3	60	120
9 m	C+ 4−	3.1	60	125	C+ 8−	3.3	60	125
	C+ 3−	1.5	60	125	C+ 7−	1.5	60	125
10 m	C+ 3–4−	1.4	60	120	C+ 7–8−	1.5	60	120
11 m	2–4+	2.3	60	130	C+ 7−	1.4	60	130
12 m	C+ 4−	3.6	60	130	C+ 8−	3.6	60	130
25 m	Parameter 1 time: 0:00–1:00, 4:00–6:00, 8:00–10:00, 13:00–15:00, 18:00–20:00, 23:00–24:00 (10 h)							
	C+ 3−	1.5	90	130	C+ 7−	1.5	90	130
	Parameter 2 time: 1:00–4:00, 6:00–8:00, 10:00–13:00, 15:00–18:00, 20:00–23:00 (14 h)							
	C+ 4−	3.6	90	130	C+ 8−	3.6	90	130
28 m	Parameter 1 time: 0:00–1:00, 4:00–6:00, 8:00–10:00, 13:00–15:00, 18:00–20:00, 23:00–24:00 (10 h)							
	C+ 3−	1.85	90	130	C+ 7−	1.85	90	130
	Parameter 2 time: 1:00–4:00, 6:00–8:00, 10:00–13:00, 15:00–18:00, 20:00–23:00 (14 h)							
	C+ 4−	3.6	90	130	C+ 8−	3.6	90	130
29 m	Parameter 1 time: 0:00–1:00, 4:00–6:00, 8:00–10:00, 12:00–15:00, 17:00–20:00, 23:00–24:00 (12 h)							
	C+ 3−	1.95	90	130	C+ 7−	1.95	90	130
	Parameter 2 time: 1:00–4:00, 6:00–8:00, 10:00–12:00, 15:00–17:00, 20:00–23:00 (12 h)							
	C+ 4−	3.6	90	130	C+ 8−	3.6	90	130
30 m	Parameter 1 time: 0:00–1:00, 4:00–6:00, 7:00–10:00, 11:00–15:00, 16:00–21:00, 23:00–24:00 (16 h)							
	C+ 3−	2.0	90	130	C+ 7−	2.0	90	130
	Parameter 2 time: 1:00–4:00, 6:00–7:00, 10:00–11:00, 15:00–16:00, 21:00–23:00 (8 h)							
	C+ 4−	3.6	90	130	C+ 8−	3.6	90	130
31 m	Parameter 1 time: 0:00–1:00, 4:00–24:00 (21 h)							
	C+ 3−	2.0	90	130	C+ 7−	2.0	90	130
	Parameter 2 time: 1:00–4:00 (3 h)							
	C+ 4−	3.6	90	130	C+ 8−	3.6	90	130
32 m	C+ 3−	2.0	90	130	C+ 7−	2.0	90	130

**Figure 1:**
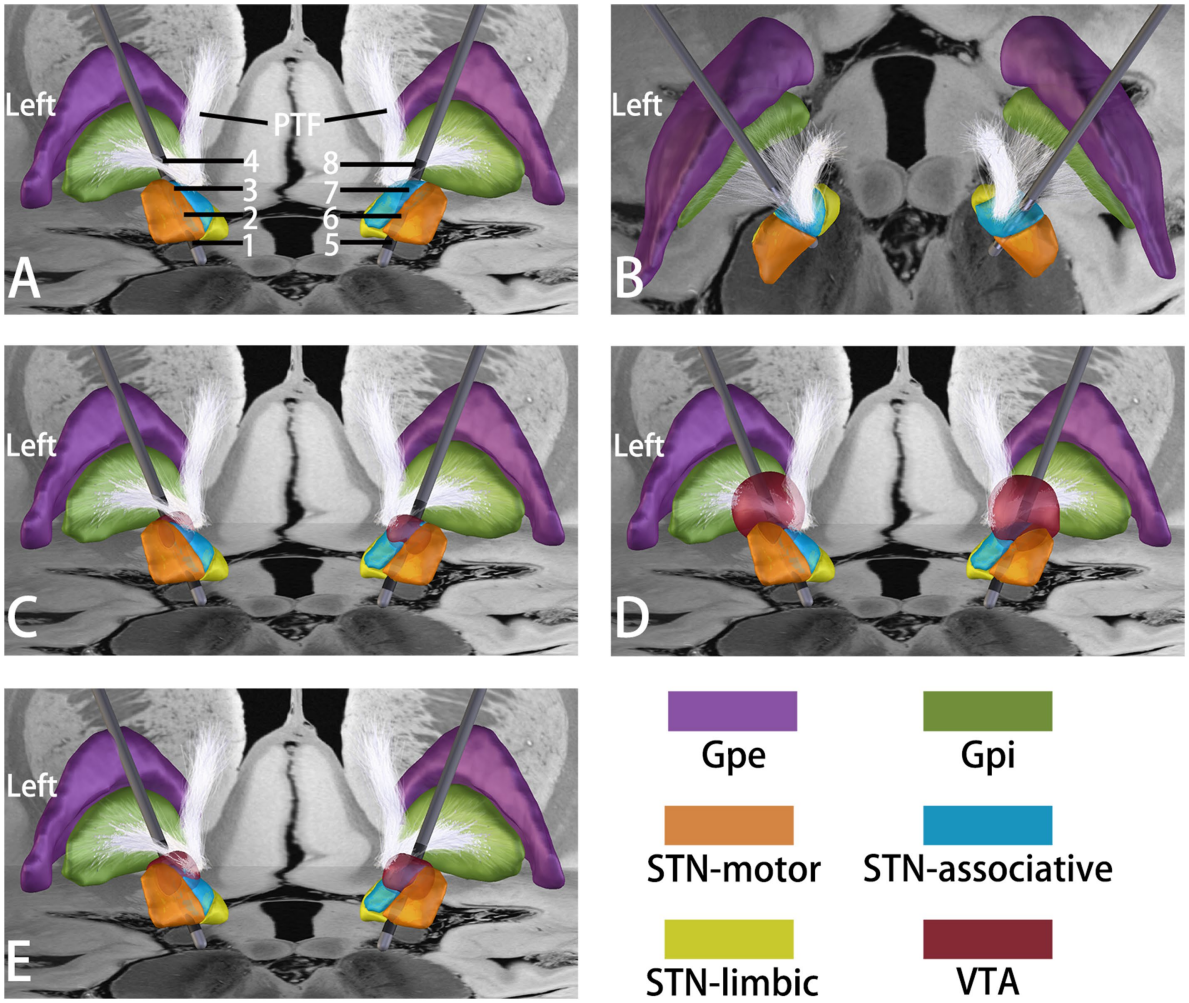
Reconstruction of the DBS electrode and estimation of the VTA by using the Lead-DBS toolbox. **(A,B)** Posterior **(A)** and superior **(B)** views of the electrode placements. The left electrode contacts are numbered 1, 2, 3, and 4, and the right electrode contacts are numbered 5, 6, 7, and 8. **(C)** Estimated VTA under the following parameters: C + 3-, C + 7-, amplitude 1.5 V, pulse width 90 μs, and frequency 130 Hz. **(D)** Estimated VTA under the following parameters: C + 4-, C + 8-, amplitude 3.6 V, pulse width 90 μs, and frequency 130 Hz. **(E)** Estimated VTA under the following parameters: C + 3-, C + 7-, amplitude 2.0 V, pulse width 90 μs, and frequency 130 Hz. STN: subthalamic nucleus, Gpe: globus pallidus externus, Gpi: globus pallidus internus, VTA: volume of tissue activated, PTF: pallidothalamic fibers.

We used the Lead-DBS toolbox (Horn et al., 2019) (version 3.2; http://www.lead-dbs.org) to reconstruct the electrode and the volume of tissue activated (VTA) (Figure 1). We found that the electrodes were positioned relatively anteriorly within the STN, with most located in the associative subregion (Figures 1A,B). In the results of the reconstructed VTA, we discovered that the VTA of the parameters initially causing SID (C + 3-, C + 7-, amplitude 1.5 V, pulse width 90 μs, and frequency 130 Hz) was primarily located in the associative subregion of the STN (Figure 1C). However, the VTA of parameter 2 during the timed-stimulation (C + 4-, C + 8-, amplitude 3.6 V, pulse width 90 μs, and frequency 130 Hz) was primarily located outside the STN covering the pallidothalamic fibers (Figure 1D). The VTA of the parameters that the patient ultimately utilized (C + 3-, C + 7-, amplitude 2.0 V, pulse width 90 μs, and frequency 130 Hz) was primarily situated in the associative subregion of the STN and partially encompassed the motor region of the STN and the pallidothalamic fibers (Figure 1E).

## Discussion

This case illustrates the efficacy of this novel phased stimulation programming strategy as a rescue therapy for SID. Approximately one-third of patients experience SID after STN-DBS (Wang et al., 2021; da Candeias Silva et al., 2025), and SID can occur in a mixture of different types of movements (Bledsoe et al., 2020) and may be perceived as the consequence of the deterioration or propagation of the underlying dystonia. However, the occurrence of SID was closely related to stimulation parameters, such as the voltage threshold and contact location, and could be resolved when stimulation was turned off. In this case report, the patient experienced SID when the stimulation voltage at a marginally more ventral contact exceeded 1.3 V. Multiple programming strategies were applied to this patient, yet none achieved a good outcome. Eventually, through timed stimulation that combined dorsal/ventral contacts and a gradual transition to single ventral contact stimulation, the patient achieved complete symptom relief without SID occurrence.

Previous studies have shown that the optimal target for STN-DBS in the treatment of Meige syndrome is located within the motor STN (Xie et al., 2024, 2025). However, it has been reported that the dyskinesia-inducing contacts are also located within the motor STN in PD patients who underwent STN-DBS (Bouthour et al., 2019). The overlap between the VTA and the motor STN has been associated with STN-DBS-induced dyskinesia (da Candeias Silva et al., 2025). In many patients, SID is transient and can be successfully managed. However, in a few patients, SID may persist despite various stimulation programs and is referred to as “brittle” STN-DBS-associated dyskinesia (Sriram et al., 2014). Previous studies indicated that in three patients (one with PD and two with obsessive-compulsive disorder) with persistent refractory SID, the occurrence of SID may have been correlated with the anteroventral and anteromedial location of the STN-DBS leads (Mulders et al., 2017; Coenen et al., 2022; Remz et al., 2023). In our patient, the electrode reconstruction results also suggested a slightly anterior location of the electrode, which was positioned within the associative subregion (Figures 1A,B). Previous findings indicated that in PD patients who underwent globus pallidus internus (GPi) DBS, SID VTAs showed significantly greater structural and functional connectivity to the associative cortex than non-SID VTAs did (Tsuboi et al., 2021). Moreover, recent evidence has indicated that SID caused by STN or GPi DBS likely shares a common pathway via subthalamopallidal connectivity (Wong et al., 2025). Therefore, in this case, the VTA predominantly overlapped with the associative STN because the anterior placement of the electrode is likely to result in greater modulation of the associative cortex, which may represent a potential mechanism underlying the patient’s persistent dyskinesia. STN-DBS-induced dyskinesias are typically observed in the dorsolateral motor STN but not in the anterior associative STN. Whether this anteriorly positioned electrode in the associative STN is associated with persistent SID requires further investigation.

SID management strategies include the use of dorsal contacts, a short pulse width, a gradual increase in the voltage, interleaved stimulation, directional stimulation, and independent current source technology (Aquino et al., 2019; Bouthour et al., 2019; Wang et al., 2021; Remz et al., 2023; da Candeias Silva et al., 2025). PD patients can also achieve resolution of SID by tapering the use of dopaminergic medications. However, in Meige syndrome patients, the resolution of SID can be achieved only through stimulation parameter programming. Therefore, for patients with severe SID, finding a set of stimulation parameters that can achieve significant improvement without causing SID is very difficult. In some patients, the dorsal relocation of STN leads or the use of GPi rescue leads was even attempted to eliminate SID. We tried various programming approaches, as mentioned above, for this patient, but the outcomes were unsatisfactory. With previous programming, the patient experienced SID at just 1.3 V when monopolar stimulation with contacts 3 and 7 was used. However, during the application of this timed stimulation regulation strategy, the patient did not experience SID when parameter 1 was used at 1.5 V. During gradually increasing the stimulation voltage, only mild SID occurred in the right arm, and it gradually disappeared. Dorsal contact stimulation has been proposed to reduce SID, likely through stimulation of pallidothalamic fibers, including the orthodromic activation of pallidothalamic fibers, which would inhibit the thalamus, and the antidromic stimulation of the GPi (Aquino et al., 2019; Goftari et al., 2020). The electrode reconstruction results in our patients revealed that the VTA of parameter 2 covered the pallidothalamic fibers (Figure 1D). Therefore, we believe that the parameter 2 stimulation of the pallidothalamic fibers may affect the SID-related neural circuits. It is likely that its delayed effect alleviates SID caused by parameter 1 stimulation. The alleviation of SID may also be related to the long-term stimulation of the pallidothalamic fibers. After 1 year of continuous parameter 2 stimulation of the pallidothalamic fibers, the patient may have developed changes in the SID-related neural circuit, which reduced SID. This hypothesis requires further investigation.

Overall, this case demonstrates that persistent refractory SID following STN-DBS in individuals with Meige syndrome can be overcome by timed stimulation that combined dorsal and ventral contacts and a gradual transition to solely ventral contact stimulation. These findings reinforce the role of pallidothalamic fiber stimulation in dyskinesia suppression. This programming method presents a viable option for persistent refractory SID. However, it is crucial to acknowledge the limitations of a single-case report which inherently restricts the generalizability of our findings. Therefore, large cohorts studies are needed to fully evaluate the efficacy of this stimulation protocol for persistent refractory SID in future.

## Data Availability

The original contributions presented in the study are included in the article/Supplementary material, further inquiries can be directed to the corresponding author.
