# Supplementary material for: Case Report: Effective management of a Meige syndrome patient with subthalamic stimulation-induced dyskinesia through timed stimulation programming of different contacts
Source: Front Hum Neurosci. 2026 Jan 22;20:1743270. doi: 10.3389/fnhum.2026.1743270 (PMC12872907; doi:10.3389/fnhum.2026.1743270)
Supplement: Supplementary file 1 [file Data_Sheet_1.docx]

**Supplementary Video 1**: Baseline dystonia symptoms before the activation of STN-DBS.

**Supplementary Video 2**: SID symptoms at 8 months after surgery **given the following parameters: C+ 3-, C+ 7-, amplitude 1.5 V, pulse width 60 μs, and frequency 130 Hz.**

**Supplementary Video 3**: Significant **alleviation of symptoms without the occurrence of SID** at 41 months after surgery **given the following parameters: C+ 3-, C+ 7-, amplitude 2.0 V, pulse width 90 μs, and frequency 130 Hz**.
